# Impact of insulin signaling and proteasomal activity on physiological output of a neuronal circuit in aging *Drosophila melanogaster*

**DOI:** 10.1016/j.neurobiolaging.2018.02.027

**Published:** 2018-06

**Authors:** Hrvoje Augustin, Kieran McGourty, Marcus J. Allen, Jennifer Adcott, Chi Tung Wong, Emmanuel Boucrot, Linda Partridge

**Affiliations:** a Max Planck Institute for Biology of Ageing, Köln, Germany; b Institute of Healthy Ageing, and GEE, University College London, London, UK; c Department of Structural and Molecular Biology, London, UK; d School of Biosciences, University of Kent, Canterbury, Kent, UK; e The Bernal Institute, University of Limerick, Limerick, Ireland

**Keywords:** Aging, Drosophila, Giant Fiber system, Gao junctions, Insulin signaling, Proteasomal activity, Recycling, Rab4, Rab11

## Abstract

The insulin family of growth factors plays an important role in development and function of the nervous system. Reduced insulin and insulin-growth-factor signaling (IIS), however, can improve symptoms of neurodegenerative diseases in laboratory model organisms and protect against age-associated decline in neuronal function. Recently, we showed that chronic, moderately lowered IIS rescues age-related decline in neurotransmission through the *Drosophila* giant fiber escape response circuit. Here, we expand our initial findings by demonstrating that reduced functional output in the giant fiber system of aging flies can be prevented by increasing proteasomal activity within the circuit. Manipulations of IIS in neurons can also affect longevity, underscoring the relevance of the nervous system for aging.

## 1 Introduction

Aging neural circuits undergo morphological and functional changes that underlie different types of behavioral impairment (Hof and Morrison, 2004). In humans, circuit-level changes during normal, nonpathological aging affect gustatory function (Iannilli et al., 2017), spatial learning (Schuck et al., 2015), working memory (Rypma and D'Esposito, 2000), and emotional states (Chen et al., 2014). In aging *Drosophila,* impairments of neural transmission in the olfactory system accompany decline in attraction behavior (Chihara et al., 2014), and in *Caenorhabditis elegans*, reduced neurotransmission drives aging-associated sensory neural activity and behavioral declines (Leinwand et al., 2015).

Insulin/insulin-like growth factor signaling (IIS) plays important physiological roles throughout the central nervous system to regulate neuronal function, metabolism, learning, and memory (D'Ercole et al., 1996). Furthermore, insulin resistance is associated with several age-related diseases, including type II diabetes and Alzheimer's disease (Bedse et al., 2015; Zick, 2005). However, protective effects of impaired IIS have been reported in a number of species during normal aging and in models of neurodegenerative diseases (Cohen et al., 2006, Gontier et al., 2015, Tsuda et al., 2010). These seemingly contradictory findings define the so called “insulin paradox” (Cohen and Dillin, 2008, Steculorum et al., 2014).

We have recently demonstrated a beneficial effect of reduced IIS on transmission through the escape response pathway of aging *Drosophila melanogaster* (Augustin et al., 2017). Systemic or circuit-specific suppression of IIS prevents the decrease in transmission speed with age by increasing membrane targeting of gap junctional proteins via small GTPases Rab4 and Rab11. Lowered IIS preserves gap junctions (GJs) in the neural circuit, resulting in a youthful functional output even in old flies (Augustin et al., 2017). Here, we have expanded these findings by further dissecting the mechanism of IIS action on the escape system function, and we have identified the proteasome as an important regulator of circuit functionality. In addition, cell culture experiments showed direct and specific impact of reduced IIS on the levels of recycling-mediating proteins Rab4 and Rab11.

The neuroendocrine axis regulates longevity and antitumorigenic response in a number of species (Tatar et al., 2003) by governing nutrient homeostasis and immune response (Fontana et al., 2010, Minor et al., 2011, Zhang et al., 2013). We have tested the impact on longevity of IIS manipulations in adult neurons and demonstrated the importance of this signaling axis in neurons in organismal aging.

## 2 Methods

### 2.1 Fly stocks and husbandry

Giant fiber (GF)-specific and ubiquitous expression was achieved with the *GAL4-UAS* system (*GAL4-*dependant upstream activator sequence) (Brand and Perrimon, 1993). The *daughterless(da)-GAL4* line (*w1118*; P13 [#8641]) was obtained from the Bloomington Drosophila Stock Center (BDSC). The dominant-negative *UAS-InR*^*dn*^ (BDSC #8252) transgene encodes an amino acid substitution in the kinase domain (K1409A) of the *Drosophila* insulin receptor (Wu et al., 2005). *UAS-InR* was also obtained from BDSC (#8262). The A307-GAL4 line was a kind gift from Dr. P. Phelan (University of Kent, Canterbury, UK); the *UAS-Rpn11* line was a gift from the lab of Dr. Masayuki Miura (University of Tokyo, Japan). To standardize genetic background, parental *GAL4* and *UAS* strains used to generate experimental and control genotypes were backcrossed to laboratory control strain white Dahomey (*w*^*Dah*^)(Wolbachia-infected) for at least six generations, beginning with an initial cross between *w*^*Dah*^ females and transgenic males, followed by five subsequent back-crosses between transgenic females and *w*^*Dah*^ males. The *w*^*Dah*^ stock was derived by incorporation of the *w*^*1118*^ mutation into the outbred Dahomey background by back-crossing. All stocks were maintained, and all experiments were conducted at 25 °C on a 12 hour:12 hour light:dark cycle at constant humidity using standard sugar/yeast/agar (SYA) medium (15 gL^−1^ agar, 50 gL^−1^ sugar, 100 gL^−1^ autolysed yeast, 100 gL^−1^ nipagin, and 3 mL^−1^ propionic acid) (Bass et al., 2007). Adult-onset neuronal expression was induced by adding mifepristone (RU486, Sigma) to the standard SYA medium at 200 μM. For pharmacological experiments, 10 μM of peripherally synapsing interneuron (Calbiochem) or 50 μM of MG132 (Sigma-Aldrich), dissolved in DMSO, was added to the standard medium. Corresponding concentrations of DMSO were added to the flies maintained on the medium without the proteasome inhibitors. For the rapamycin experiment, 5 μm of rapamycin was added to the chemically defined (holidic) medium using the previously published protocol and recipe (Piper et al., 2014); this concentration has been shown to significantly reduce egg-laying capacity (Piper et al., 2014). For all experiments, including life span experiments, flies were reared at standard larval density, and eclosing adults were collected over a 12 hours period. Flies were mated for 48 hours before separating females from males.

### 2.2 Electrophysiology

Preparation of flies and recordings from the giant fiber system (GFS) of adult flies were performed as described by Allen et al. (Allen et al., 1999); a method based on those described previously (Gorczyca and Hall, 1984, Tanouye and Wyman, 1980). Briefly, flies were anaesthetized by cooling on ice and secured in wax placed inside a small Petri dish, ventral side down, with the wings held outward in the wax to expose lateral and dorsal surfaces of the thorax, and the proboscis pulled outward and pushed into the wax so that the head lied slightly forward and down on the surface. A tungsten earth wire placed in the posterior end of the abdominal cavity served as a ground electrode. Extracellular stimulation of the GFs was achieved by placing two electrolytically (NaOH) sharpened tungsten electrodes through the eyes and into the brain (the supra-oesophageal ganglion) to deliver a 40 V pulse for 0.03 ms using a Grass S48 stimulator (a stimulus between 40–60 V will generate a response). The stimulating and ground electrodes do not need to be replaced during a recording session. Threshold for the short-latency, direct excitation for GF stimulation was previously demonstrated to be a pulse of ∼10–20 V for 0.03 ms (Engel and Wu, 1996, Tanouye and Wyman, 1980).

Intracellular recordings were made following GF stimulation from the tergo-trochanter muscle (TTM) and contralateral dorsal longitudinal muscle (DLM) using glass micropipettes (resistance: 40–60 MΩ). The possibility that descending neurons other than the GFs might be simultaneously activated, leading to a possible TTM or DLM response, was previously excluded (Allen et al., 1999). The glass electrodes were filled with 3M KCl and placed into the muscle fibers through the cuticle. Responses were amplified using Getting 5A amplifiers (Getting Instruments, USA), and the data were digitized using an analogue-digital Digidata 1320 and Axoscope 9.0 software (Molecular Devices, USA). For response latency recordings, at least 5 single stimuli were given with a 5 seconds rest period between each stimulus; measurements were taken from the beginning of the stimulation artefact to the beginning of the excitatory postsynaptic potentials (i.e., muscle depolarization). The signals were amplified and stored on a PC with pCLAMP software (Molecular Devices, USA). Analysis was performed on the PC using pCLAMP and Microsoft Excel 2010 software (Microsoft, USA).

### 2.3 Life span experiments

Flies in life span experiments were reared at standard larval density, and eclosing adults were collected over 12 hours periods. Newly eclosed flies were transferred to new bottles without anesthesia and allowed to mate for 48 hours (“once mated”). Sexes were separated by brief CO_2_ exposure, and the female flies were transferred into experimental vials. Flies were maintained in vials on standard SYA medium at a density of ten flies per vial and transferred to new vials every 2–3 days by CO_2_ anesthesia and scored for deaths.

### 2.4 Cell culture

Retinal pigment epithelial-1 cells (ATCC CRL-4000) were maintained at 37 °C, 5% CO2, in a complete medium (DMEM:F12 HAM [1:1 v/v] [Sigma] supplemented with 10% fetal bovine serum [Life Technologies], 0.5% [w/v] sodium bicarbonate [Sigma], 2 mM glutamate, antibiotic-antimycotic [Sigma], and 20 ng/mL hygromycin). Cells were regularly tested for mycoplasma contamination. For all assays, retinal pigment epithelial-1 cells were seeded in appropriate culture dishes (approximately 5x10^6^ cells were seeded per 10-cm plastic tissue culture dish) and grown as monolayers for four days in complete medium (containing insulin from fetal bovine serum). Cells were then treated as follows: IIS was lowered on 1-hour treatment with an insulin receptor (IR) and insulin-like growth factor-1 receptor dual inhibitor (BMS 536924 used at 5 μM, Tocris 4774, referred to as “+ IR inhib”) or on removal of insulin (“- insulin”) by incubating the cells in serum-free medium (containing glucose but no insulin) for 11 hours. IIS was elevated by addition of 1 μM insulin (MP Biomedicals) for 1 hour.

### 2.5 Immunoblot measurments of total cellular Rab protein levels

Treated cells were collected in Laemmli sample buffer, sonicated and boiled. Samples were run on NuPage 4%–12% Bis-Tris gel (Life Technologies), transferred to PVDF membranes (Perkin Elmer), blocked in 5% skimmed milk and incubated successively with primary and secondary-HRP coupled antibodies, and finally visualized with ECL (Thermo Scientific) or Luminata Crescendo (Millipore) HRP reagents depending on the strength of the signals. Signals were captured on Amersham Hyperfilm ECL (GE Healthcare), developed using a Xograph Compact X4 film developer and analyzed using ImageJ software (National Institutes of Health, USA). Signals used for quantifications were captured at a pre-saturation intensity. Results are derived from triplicate biological repeats and represent signals that were normalized to a glyceraldehyde 3-phosphate dehydrogenase (GAPDH) loading control and to the signals from resting cells. Antibodies used were mouse anti-Rab4 (BD Biosciences 610888), mouse anti-Rab5 (BD Biosciences 610282), rabbit anti-Rab7 (Cell Signaling Technologies D95F2), rabbit anti-Rab8 (Cell Signaling Technologies D22D8), rabbit anti-Rab11 (Life Technologies, 715300), mouse anti-GAPDH (Santa Cruz 0411), and rabbit anti-GAPDH (Cell Signaling Technologies 14C10).

## 3 Statistical analyses

Statistical analyses were performed using GraphPad Prism 5 software (GraphPad Software Inc, USA). A two-way analysis of variance test was used to perform (age × genotype) interaction calculations. For other comparisons between two or more groups, a one-way analysis of variance followed by a Tukey-Kramer post hoc test was used. In all instances, *p* < 0.05 is considered to be statistically significant (*p < 0.05; **p < 0.01; ***p < 0.001). All error bars denote the standard error of the mean. The log-rank test was used to calculate *p* values and compare survival distributions between pairs of cohorts. Microsoft Excel was used for these analyses.

## 4 Results

**Fig. 1:**
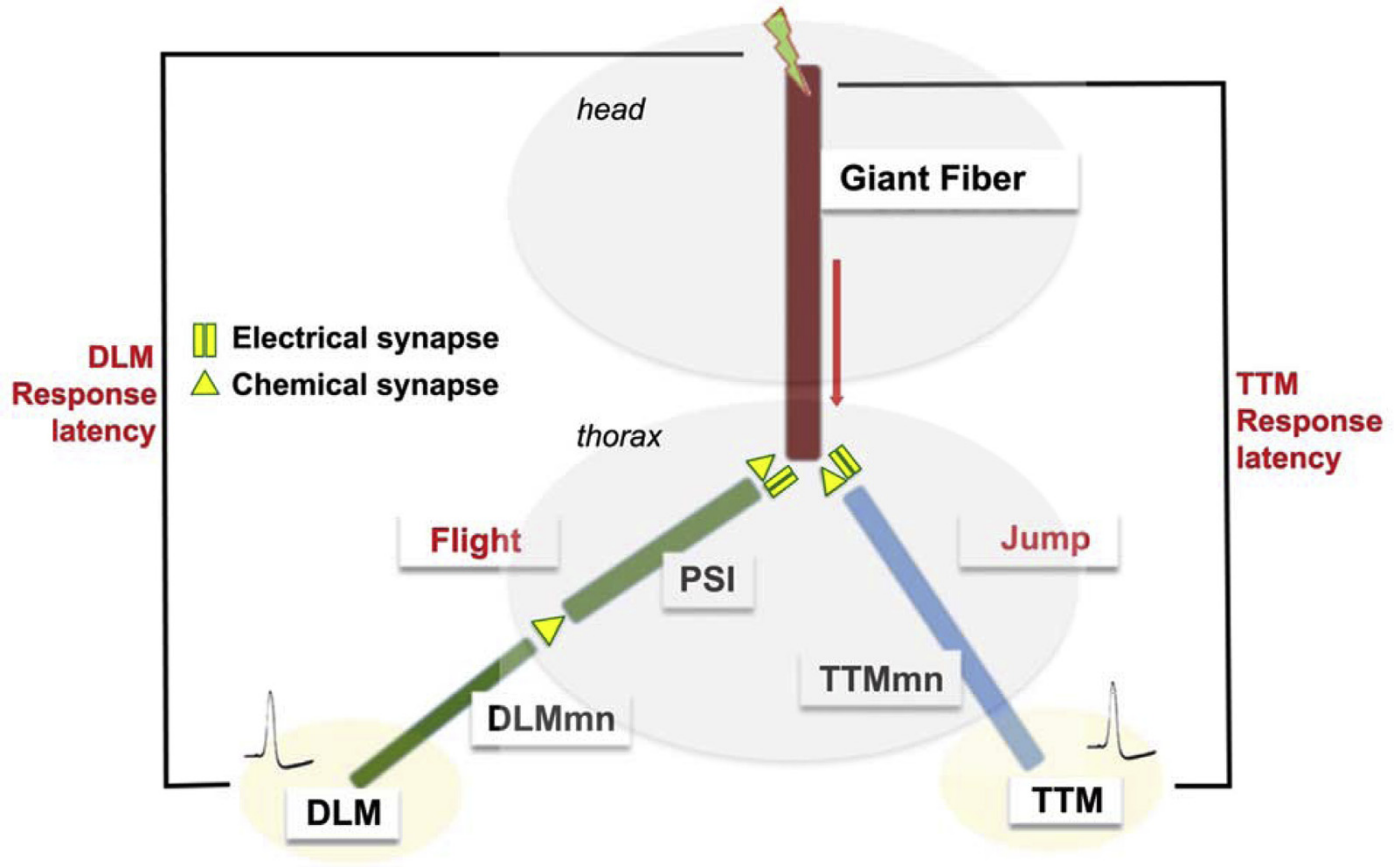
Schematic representation of the *Drosophila* escape response pathway. The GFS consists of two descending giant fiber (GF) interneurons that originate in the brain, and downstream neurons and muscles. In the thoracic region, the GFs form mixed (chemical and electrical) synapses with the peripherally synapsing interneuron (PSI), which in turn forms cholinergic synapses with the motoneuron (DLMn) innervating dorsal longitudinal flight muscles. The GFs also directly connect (via mixed synapses) with the TTMn, the motoneuron that innervates the tergo-trochanteral, or jump, muscle. Electrical brain stimulation (green lightning bolt) activates the giant fiber interneurons, and the two output pathways can be monitored by recording from the two muscles (depolarization spikes). Abbreviations: GFS, giant fiber system; DLM, dorsal longitudinal muscle; TTM, tergo-trochanteral muscle. (For interpretation of the references to color in this figure legend, the reader is referred to the Web version of this article.)

Escape responses in many invertebrate and lower vertebrate species are mediated by giant nerve fibers (Allen et al., 2006). First described in the squid ganglion (Young, 1936), the simple “Giant Fiber” circuits are a convenient system for studying neural development and function. In the fruit fly *Drosophila*, the GFS comprises a small number of anatomically and functionally well-defined neurons (Trimarchi and Schneiderman, 1994) amenable to molecular, genetic, electrophysiological and behavioral studies. The GFS is composed of electrical, chemical and mixed synapses, with transmission via *Shaking-B*-encoded GJs responsible for the predominantly ‘electrical’ character of the circuit (Allen and Murphey, 2007, Phelan et al., 1996, Trimarchi and Murphey, 1997). The circuit mediates flight initiation following either visual or olfactory stimuli (Trimarchi and Schneiderman, 1995a, Trimarchi and Schneiderman, 1995b), through activation of both flight (DLM) and jump (TTM) muscles (Fig. 1). We measured the speed of signal propagation through the GFS by directly stimulating the GF cell bodies in the fly brain and recording “response latencies” from the downstream muscles (TTM and DLM) (Allen and Godenschwege, 2010, Augustin et al., 2011). Increased response latency indicates slower transmission and diminished circuit function.

**Fig. 2:**
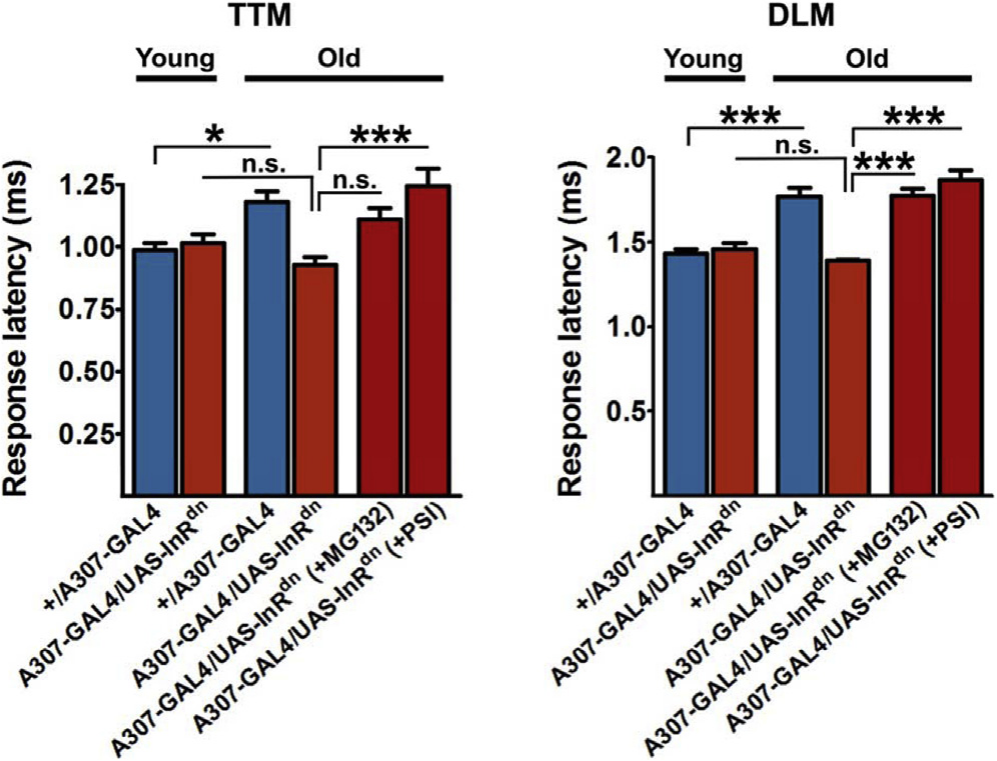
Proteasomal inhibition abolishes the effect of reduced IIS. Reduced IIS (*A307-GAL4/UAS-InR*^*dn*^) prevented age-related increase in response latency observed in the control genotype (*+/A307-GAL4*) (bars 1-4 in each panel [Augustin et al., 2017]). Proteasome inhibitors MG132 and PSI fed to adult *A307-GAL4/UAS-InR*^*dn*^ flies starting one day after their emergence from pupal cases (the two rightmost bars in each panel) abolished the effect of reduced IIS. N = 6–9 per genotype/condition/age. One-way ANOVA: *p < 0.05; ***p < 0.001. Error bars represent SEM. Abbreviations: ANOVA, analysis of variance; DLM, dorsal longitudinal muscle; IIS, insulin and insulin-growth-factor signaling; n.s., not significant; PSI, peripherally synapsing interneuron; SEM, standard error of the mean; TTM, tergo-trochanteral muscle.

**Fig. 3:**
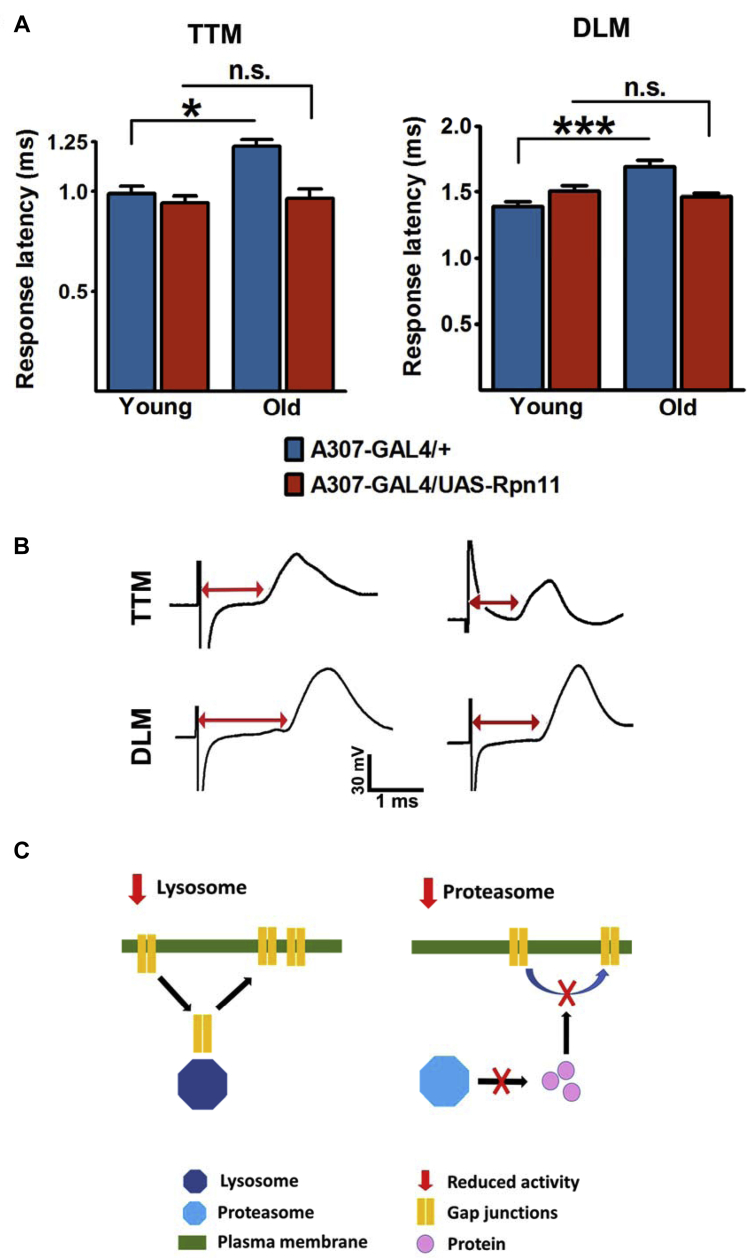
Overexpression of a proteasome regulatory (19s) subunit prevented age-related functional decline in the GFS. (A) Response latency measured in the TTM (left) and DLM (right) branch of the circuit. Rpn11 encodes one of the “lid” subunits of the proteasome 19S particle, involved in selecting and unfolding proteins targeted for degradation by the 20S core/catalytic particle. N = 4–7 per genotype per age. The genotype-age interaction (two-way ANOVA) was significant: TTM: *p* value = 0.0335; DLM: *p* value = 0.0012. One-way ANOVA (Tukey's test): *p < 0.05; ***p < 0.001. Error bars represent SEM. (B) Representative TTM and DLM traces from old flies from (A). Red arrows indicate longer response latencies in control (left) and shorter in Rpn11-overexpressing animals (right). (C) Model for the effect of reduced lysosomal and proteasomal activity on the targeting of GJ proteins to the plasma membrane (see main text). Abbreviations: ANOVA, analysis of variance; DLM, dorsal longitudinal muscle; GFS, giant fiber system; GJ, gap junction; n.s., not significant; SEM, standard error of the mean; TTM, tergo-trochanteral muscle. (For interpretation of the references to color in this figure legend, the reader is referred to the Web version of this article.)

A dominant-negative form of the insulin receptor (Poirier et al., 2008) can be used to attenuate IIS in flies, and diminished IIS in the GFS prevents age-associated increase in response latency (bars 1-4 in Fig. 2 [Augustin et al., 2017]). Escape response circuit-specific IIS reduction was achieved using the *A307-GAL4* line, which drives expression strongly in the GFs and, to a lesser extent, in the TTM and DLM motoneurons and peripherally synapsing interneurons (Allen et al., 1998, Phelan et al., 1996). Pharmacological suppression of proteasomal activity neutralized the beneficial effect of reduced IIS (Augustin et al., 2017) on the circuit function in old flies (Fig. 2). Although reduced IIS does not ameliorate the age-associated reduction in chymotrypsin-like peptidase activity of the proteasome in the fly nervous system (Augustin et al., 2017, Tonoki et al., 2009), these results suggest that proteasomal activity is required for the prevention of functional decline in the GFS. To further investigate the effect of the proteasome on the GF circuit, we overexpressed Rpn11, a component of the proteasomal regulatory subunit, in the neurons of the GFS. Rpn11 is one of the “lid” subunits in the 19S proteasomal regulatory particle and was previously reported to suppress the age-related decline in proteasomal activity and progression of the polyglutamine-induced neurodegenerative phenotype in aging flies (Tonoki et al., 2009). In line with these findings, *Rpn11* overexpression prevented the age-related functional decline in the GFS (Fig. 3A and B). Together, these results demonstrate the importance of the proteasome on the function of the escape response pathway in aging flies and suggest increased proteasomal activity as a way to improve age-related functional decline of neural circuits. Previously, we demonstrated a correlation between GF transmission and synaptic levels of gap junctional proteins (Augustin et al., 2017). As inhibition of the lysosome increases the density of GJ aggregates (Augustin et al., 2017), the results presented here suggest that reduction of proteasomal activity has a negative effect on their synaptic accumulation (Fig. 3C). Reduced proteasomal activity likely compromises proteostasis of other proteins, thereby indirectly impairing recycling and membrane targeting of gap junctional proteins.

**Fig. 4:**
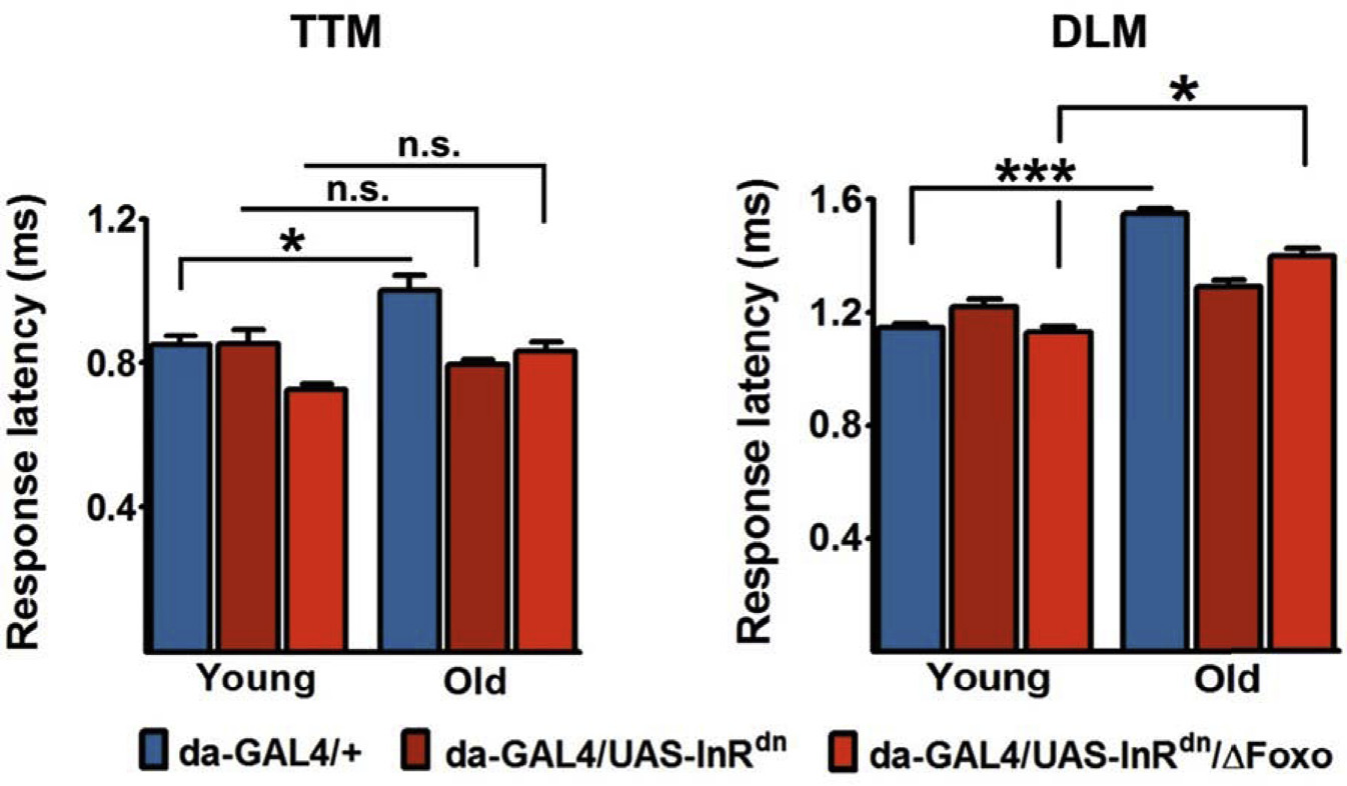
dFoxo partially mediates the effect of reduced IIS on response latency. Deletion of Foxo did not abolish the effect of attenuated IIS in the TTM branch (left), but it reversed the action of lowered IIS in the DLM (right) branch of the giant fiber circuit. N = 4–10 per genotype per age. One-way ANOVA (Tukey's test): *p < 0.05; ***p < 0.001. Error bars represent SEM. Abbreviations: ANOVA, analysis of variance; IIS, insulin and insulin-growth-factor signaling; DLM, dorsal longitudinal muscle; n.s., not significant; SEM, standard error of the mean; TTM, tergo-trochanteral muscle.

To further examine how attenuated IIS affects GFS function, we measured response latencies in flies with ubiquitously reduced IIS (*da-GAL4/UAS-InR*^*dn*^) in the presence or absence of Foxo, a well-described downstream mediator of IIS action in flies and mammals (Partridge and Bruning, 2008). Interestingly, deletion of *dFoxo* had no effect on the ability of reduced IIS to maintain TTM response latencies in old flies at the youthful level but reversed the effect of low IIS on the DLM branch of the circuit (Fig. 4). These results indicate a complex role for IIS in regulating the GFS physiology in aging flies. Unlike the more “electrical” nature of the TTM branch, the DLM part of the circuit is dominated by chemical synaptic connections (Fig. 1 [Allen et al., 2006]); it is therefore intriguing that Foxo may have role specifically as a regulator of chemical neurotransmission downstream of IIS.

**Fig. 5:**
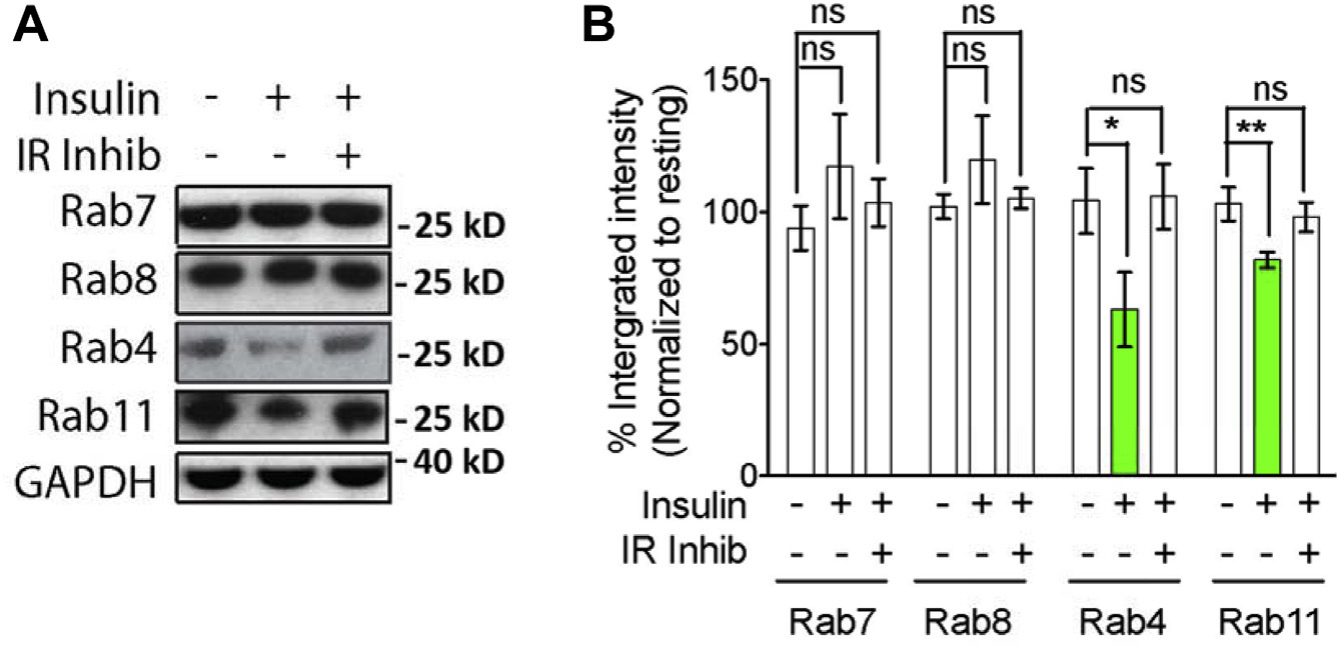
IIS negatively regulates recycling mediating Rab proteins. (A) Representative immunoblots of the levels of the indicated Rab proteins and GAPDH (loading control) from RPE-1 cells treated as indicated with insulin or insulin and IR Inhib. (B) Quantification histograms of the immunoblots from (A). Levels were normalized to respective GAPDH amounts and Rab levels in resting control cells. N = 3 independent experiments. One-way ANOVA (Tukey's test): *p < 0.05; **p < 0.01. Error bars represent SEM. Abbreviations: ANOVA, analysis of variance; IIS, insulin and insulin-growth-factor signaling; IR, insulin receptor; n.s., not significant; RPE-1, retinal pigment epithelial-1; SEM, standard error of the mean.

We have previously shown that GJs are regulated at the protein level in response to acute and long-term IIS (Augustin et al., 2017). Elevated IIS induces the targeting of GJ proteins to lysosomes and degradation, thereby decreasing their cell surface assembly. This phenotype could be suppressed by enhancing endosomal recycling by overexpressing wild-type or constitutively active forms of Rab4 or Rab11, mimicking IIS attenuation (Augustin et al., 2017). We therefore asked if the endosomal recycling machinery itself could similarly be regulated by IIS. Strikingly, Rab4 and Rab11 were present at significantly lower levels in mammalian cells on insulin addition, indicating a direct impact of IIS on the levels of these recycling Rab proteins (Fig. 5). Interestingly, this effect was specific for the recycling Rabs, as Rab7 and 8 were unaffected. Cumulatively, these results show that IIS has a marked impact on protein levels at multiple junctures in the cell including the protein degradative and recycling machinery.

**Fig. 6:**
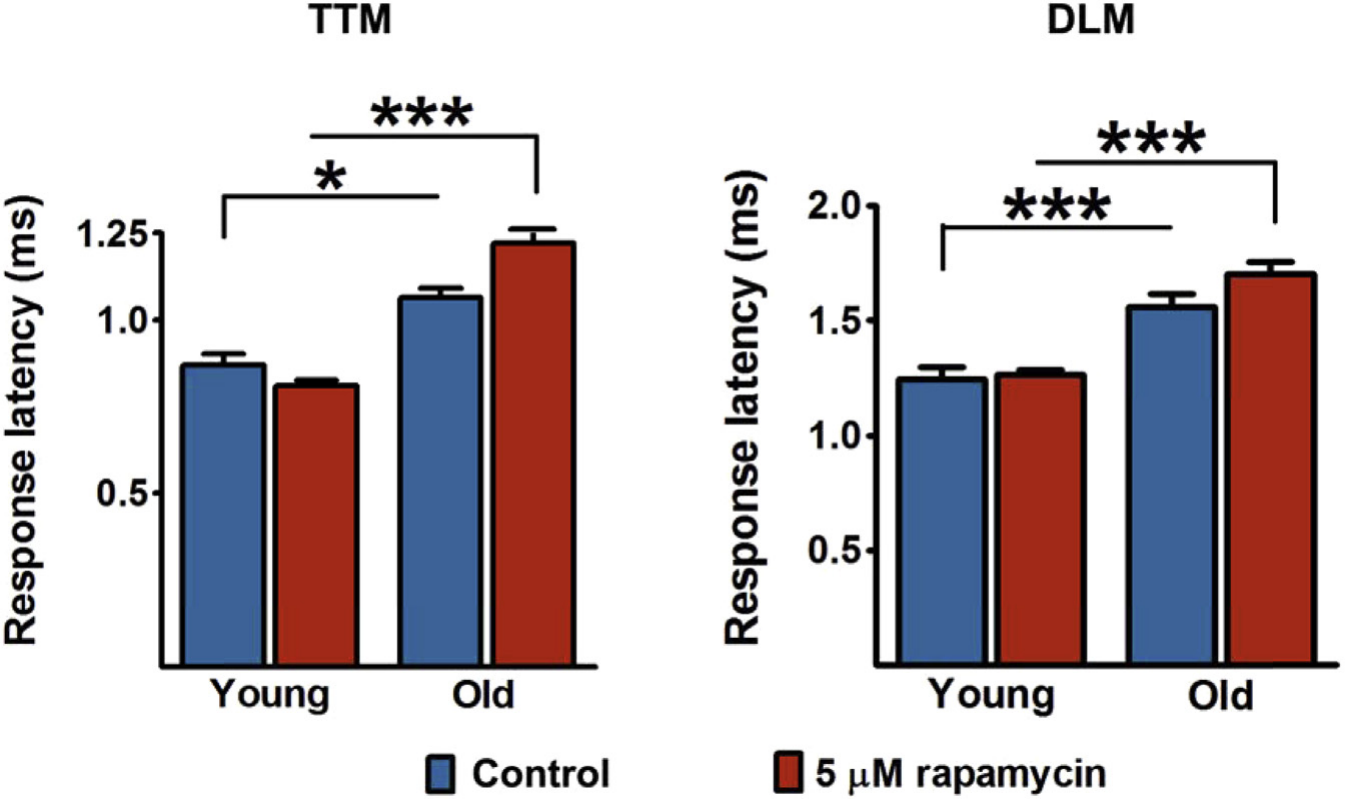
Rapamycin does not reverse the effect of age on TTM and DLM response latency in wild-type (*w*^*Dah*^) flies. (Left): TTM branch. (Right): DLM branch. N = 6–8 per condition/age. One-way ANOVA (Tukey's test): *p < 0.05; ***p < 0.001. Error bars represent SEM. Abbreviations: ANOVA, analysis of variance; DLM, dorsal longitudinal muscle; SEM, standard error of the mean; TTM, tergo-trochanteral muscle.

Together with IIS, the mammalian target of rapamycin signaling network plays a key role in regulating metabolism and in life span in *Drosophila* and other species (Wei et al., 2013). Inhibition of the fly TORc1 complex by rapamycin (Bjedov et al., 2010) did not affect response latency in aging flies (Fig. 6), indicating a specificity of lowered IIS action on GFS function.

**Fig. 7:**
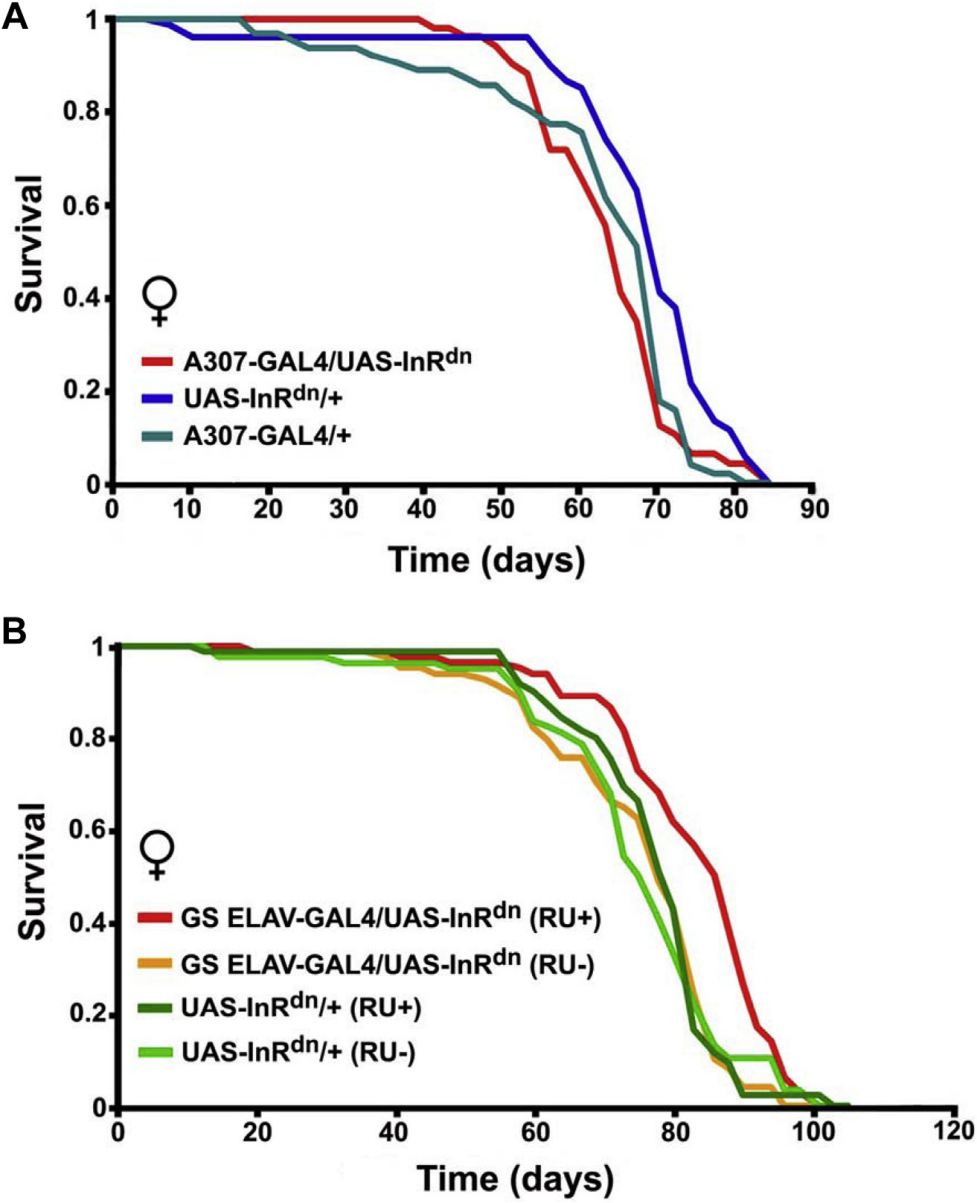
Effect on life span of attenuated IIS in neurons. (A) GFS-specific overexpression of a dominant-negative form of the *Drosophila* insulin receptor had no effect on median or maximum life span. Median life span (days): *A307-GAL4/+* (68); *UAS-InR*^*dn*^*/+* (68); *A307-GAL4/UAS-InR*^*dn*^ (64). (B) Reduced IIS in adult neurons extended median life span. Median life span (days): *UAS-InR*^*dn*^*/+*, RU- (76); *UAS-InR*^*dn*^*/+*, RU+ (78); *GS ELAV-GAL4/UAS-InR*^*dn*^*,* RU- (76); *GS ELAV-GAL4/UAS-InR*^*dn*^, RU+ (86). *p*-value for significance between *GS ELAV-GAL4/UAS-InR*^*dn*^ (RU+) and other genotypes: 0.05. Abbreviations: GFS, giant fiber system; IIS, insulin and insulin-growth-factor signaling.

Reduced IIS in either the GFS or adult nervous system abolishes the prolonged response latencies seen in aged wild-type or control flies, with IR up-regulation exacerbating the phenotype (Augustin et al., 2017). As improved function (“health span”) and life span are frequently correlated in various species, (see e.g., [Castillo-Quan et al., 2016, Martin-Montalvo et al., 2013]) we assessed the effect of IIS manipulations in the GFS, or adult neurons, on longevity. We overexpressed the dominant-negative variant of the *Drosophila* insulin receptor using the inducible *GS ELAV-GAL4* nervous system driver (Osterwalder et al., 2001). While GFS-specific reduction of IIS had no effect on life span (Fig. 7A), likely due to the relatively small overall size of the circuit, IIS attenuation in all adult neurons using the inducible driver extended median life span (Fig. 7B), implicating the adult nervous system as playing a key role in overall health during aging in flies.

## 5 Discussion

Proteasomal activity in the brain of *D. melanogaster* declines with age (Augustin et al., 2017, Tonoki et al., 2009), in line with reports of age-related alterations in proteasome-mediated proteolysis in the aging mammalian brain (Keller et al., 2004) and in neurodegenerative diseases (Keller et al., 2000). Here, we show that proteasomal up-regulation can maintain neurotransmission through the escape response circuit in aging flies, and that basal levels of proteasomal activity are required for the beneficial effect of attenuated IIS on age-related circuit function. Consistent with the effect of proteasomal activation in other species (Chondrogianni et al., 2015a, Chondrogianni et al., 2015b), overexpression of *Rpn11* suppresses the accumulation of ubiquitinated proteins in the fly brain, likely by promoting 26S proteasome assembly (Tonoki et al., 2009). Because of the predominantly postmitotic status of neurons, the nervous system is particularly prone to age-associated increase in oxidative stress (Keller and Mattson, 1998). Together with a decrease in antioxidant capacity during normal aging (Golden et al., 2002), these changes cause the accumulation of damaged and misfolded proteins in aging neuronal cells (Keller et al., 2004). In addition to the lysosome and autophagy, the proteasomal system is critical for degradation and disposal of abnormal proteins, and decline in proteasomal function (Augustin et al., 2017, Tydlacka et al., 2008) may further increase the buildup of aberrant protein aggregates (Graham and Liu, 2017). These age-dependant changes likely contribute to morphological and physiological defects in neurons, such as their ability to maintain synaptic and cytoskeletal integrity and regulate intracellular signaling and protein trafficking (Keller et al., 2004). Considering its wide role in protein homeostasis and quality control (Cajigas et al., 2010, Malgaroli et al., 2006), the proteasomal system is likely to affect many components of the neuronal machinery. Reduced proteasomal activity will therefore inevitably lead to compromised cellular health and impaired synaptic function (Cajigas et al., 2010). Indeed, both degradation and synthesis of synaptic proteins are disrupted following pharmacological inhibition of the proteasomal machinery (Hakim et al., 2016). Rapamycin, a well-described activator of autophagy that extends life span in flies (Bjedov et al., 2010), had no effect on the GFS function (this study), further underscoring the requirement for proteasomal degradation, presumably of proteins other than those involved in GJs and the recycling machinery, in the maintenance of circuit functionality.

Previously, we showed that beneficial effect of reduced IIS on the neurophysiological output in aging flies requires the presence of the recycling machinery (Augustin et al., 2017). Our experiments in cultured cells presented here also identified insulin as a negative regulator of the levels of the small GTPases Rab4 and Rab11, suggesting a complex interplay between IIS and the trafficking pathways mediating endosomal recycling. Since insulin/insulin-like growth factor receptors are themselves recycled through the recycling machinery (Romanelli et al., 2007), this finding suggests a novel mechanism of feedback in IIS itself. The functional consequences of these interactions in healthy and pathological organismal aging remain to be explored.

In the context of GJs, increased endocytic recycling activity through Rab4 and Rab11 (or reduced insulin signaling) rescues internalized GJ proteins from terminal degradation in the lysosome, promoting their accumulation in the plasma membrane.

IIS are evolutionarily conserved growth-promoting pathways that play critical roles in both developing and adult brains (D'Ercole et al., 1996, Nieto-Estevez et al., 2016). Mutations that reduce IIS, however, can dramatically extend life span in a number of species (Fontana et al., 2010). The nervous system is one of the most important sites for life span extension by IIS (Broughton and Partridge, 2009, Wrigley et al., 2017). For example, brain-specific knockout of either the IR substrate-2 (an intracellular mediator of insulin action) or insulin-like growth factor-1 receptor has been reported to extend life span in mice (Kappeler et al., 2008, Taguchi et al., 2007); in the nematode *C. elegans*, the nervous system is also critical for increased longevity by IR inactivation (Apfeld and Kenyon, 1998, Wolkow et al., 2000). Here, we corroborated the importance of neuronal control of life span by identifying that IIS activity only in the adult nervous system reduces longevity in *Drosophila*. Recently, reported life span extension by means of reduced IIS in the nervous system throughout life (Ismail et al., 2015) could therefore be due to adult-only IIS suppression. Further studies are required to reconcile the seemingly contradictory effects of IIS on organismal function and longevity.

## Disclosure statement

The authors have no actual or potential conflicts of interest.
